# InAs/GaAs Quantum Dot Dual-Mode Distributed Feedback Laser Towards Large Tuning Range Continuous-Wave Terahertz Application

**DOI:** 10.1186/s11671-018-2674-3

**Published:** 2018-09-04

**Authors:** Qi-zhu Li, Yuan-qing Huang, Ji-qiang Ning, Cheng Jiang, Xu Wang, Hong-mei Chen, Xiao Li, Rui-ying Zhang, Kai Zhang, Jia-hua Min, Yong Peng, Zi-yang Zhang

**Affiliations:** 1 Key Laboratory of Nanodevices and Applications, Suzhou Institute of Nano-Tech and Nano-Bionics, Chinese Academy of Sciences, Suzhou, 215123 People’s Republic of China; 2 Lab of Magnetism and Magnetic Materials of the Ministry of Education, School of Physical Sciences and Technology, Electron Microscopy Centre of Lanzhou University, Lanzhou University, Lanzhou, 730000 People’s Republic of China; 3 Vacuum Interconnected Nanotech Workstation, Suzhou Institute of Nano-Tech and Nano-Bionics, Chinese Academy of Sciences, Suzhou, 215123 People’s Republic of China; 4 i-Lab, Suzhou Institute of Nano-Tech and Nano-Bionics, Chinese Academy of Sciences, Suzhou, 215123 People’s Republic of China; 5 School of Materials Science and Engineering, Shanghai University, 200444 Shanghai, People’s Republic of China

**Keywords:** Quantum well, wire, and dot devices, Distributed feedback, Terahertz imaging, Lasers, Semiconductor lasers, Optoelectronics

## Abstract

In this paper, a laterally coupled distributed feedback (LC-DFB) laser based on modulation p-doped multiple InAs/GaAs quantum dot (QD) structures has been fabricated. The device exhibits a high side-mode suppression ratio (SMSR) of > 47 dB and a high thermal stability of dλ/dT = 0.092 nm/K under continuous-wave (CW) operation, which is mainly attributed to the high material gain prepared by modulation p-doping and rapid thermal annealing (RTA) process, and the significantly reduced waveguide losses by shallow-etched gratings and its close proximity to the laser ridge feature in the LC-DFB laser. With this superior performance of the DFB laser, the wide tunable dual-wavelength lasing operation has been obtained by delicately defining different periods for the grating structures on the two sides of the laser ridge or combining the reduced laser cavity length. The wavelength spacing between the two lasing modes can be flexibly tuned in a very wide range from 0.5 to 73.4 nm, corresponding to the frequency difference from 0.10 to 14 THz, which is the largest tuning range by the utilization of single device and hence providing a new opportunity towards the generation of CW THz radiation.

## Background

Distributed feedback (DFB) lasers are technologically significant for their wide range of applications in long-distance fiber optical communication and terahertz (THz) radiation due to their narrow emission spectrum and stabilized emission wavelength [1–3]. Great efforts and various attempts have been made in the past decade to pursue high-performance DFB lasers, and quantum dot (QD)-based DFB lasers have exhibited advantageous performances such as low threshold current, high quantum efficiency, broadband wavelength tuning range, and high-temperature stability over commercial quantum well-based devices [4–6]. The modulation p-doping in quantum dot laser structures has been demonstrated as an effective method to further improve the QD laser performance including the temperature stability [7] and high-speed modulation characteristics [8] due to the significantly enhanced ground state (GS) gain. Moreover, it has also been found that rapid thermal annealing (RTA) is another efficient way to optimize the material quality and optical properties of the QD assemblies, owing to the reduction of point defects and dislocations that are produced during the epitaxy growth. The conventional fabrication process of a DFB laser usually requires two steps of high-quality epitaxial growth [9]. Stubenrauch et al. reported the fabrication of a 1.3-μm QD DFB laser which shows high static and dynamic performance; however, after the fabrication of a Bragg grating structure and epitaxy growth of bottom cladding layer and active region, a metalorganic vapor chemical deposition (MOCVD) epitaxial re-growth step is required to complete the whole laser structure leading to many complex and uncertain factors [1]. To avoid the re-growth process, Goshima et al. proposed a QD-based laterally coupled distributed feedback (LC-DFB) laser structure which was realized by deeply etching the grating vertically into the ridge waveguide, but low slope efficiencies below 0.03 W/A and small side-mode suppression ratio (SMSR) of 20 dB were observed due to large waveguide losses [10]. The waveguide losses are mainly from the deep etching process, by which the high-quality and uniform grating structure is very difficult to realize due to the technical issues of high aspect ratio (normally 20:1) requirement in either dry etching or wet etching process [11]. So, in order to realize a super high-performance DFB laser, it is necessary to trace a way to combine the optimized QD active region with improved device waveguide structure together.

Terahertz (THz) frequency radiation sources have attracted considerable attention for their prosperous medical, agriculture, environment, and security applications [12, 13] and that frequency-tunable continuous-wave (CW) operation of the THz radiation source with compact size and low cost is especially desired. Recently, various semiconductor dual-mode lasers have been studied for the aim of developing an optical beat source for THz photomixing. Broad frequency tuning has been demonstrated by using external-cavity lasers which emit two lines of different wavelengths simultaneously [14, 15]. However, the mechanical moving parts in the external-cavity laser system are neither convenience nor stable for wavelength tuning. CW THz signals can also be generated by using two independent DFB laser beams of slightly different frequencies. This technique has emerged as an excellent choice to generate THz radiation benefitting from the very narrow emission spectrum and stabilized emission wavelength of DFB laser diodes [3, 16–18]. Besides those reported configurations for THz photomixing, the simultaneous emission of two tunable laser lines from a single DFB laser cavity is very appealing due to its compactness, high-temperature stability, and high spectral quality [3, 19].

In this work, the multiple InAs/GaAs QD laser structures were grown by molecular-beam epitaxy (MBE), and p-type modulation doping was applied to the QD active region. After epitaxy growth, the QD samples were treated by a post-growth annealing process. To avoid the overgrowth step and reduce the aspect ratio in grating etching, the LC-DFB laser was fabricated with shallow-etched gratings. The shallow-etched LC-DFB lasers based on the p-doped QDs exhibit a high differential efficiency of 0.2 W/A, a large SMSR of 47 dB, and a high thermal stability dλ/dT of 0.092 nm/K. In addition, dual-mode lasing was successfully obtained with the LC-DFB lasers by fabricating two sets of gratings of different periods, and the lasing wavelengths can be simply manipulated by delicately modifying the grating periods, which enable a large range tuning of the frequency difference of the two lasing modes from 0.10 to 14 THz. Our work demonstrates the promise applications of QD-based LC-DFB lasers for long-distance fiber-optic communication and CW THz radiation sources.

## Methods

### Preparation and Characterization of Materials

**Fig. 1:**
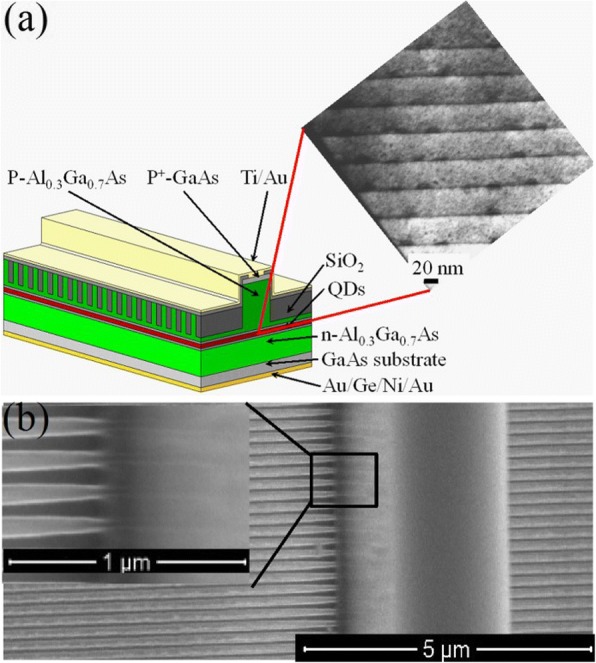
Schematic diagram and morphology of InAs/GaAs QDs LC-DFB laser structure. **a** Schematic diagram of InAs/GaAs QD LC-DFB laser structure. Inset: cross-sectional TEM image of the QD active layer structure. **b** The top view of the SEM image of LC-DFB laser structure with first-order grating. Inset: enlarged SEM image focus on the joint between the grating and ridge waveguide

The InAs/GaAs QD laser structures were grown on Si-doped GaAs (100) substrates by a MBE system. The active region of the laser structure is eight stacks of QD layer separated by GaAs barriers of 33 nm in thickness. Each QD layer comprises 2.7 ML InAs covered with a 6-nm-thick InGaAs strain-reducing layer. And the whole active region is sandwiched by the cladding layers of the lower ~ 2800 nm n-Al_0.3_Ga_0.7_As and the upper ~ 1800 nm p-Al_0.3_Ga_0.7_As. The deposition of the InAs at a growth temperature of 510 °C and a growth rate of 0.01 ML/s. The modulation p-doping with Be was conducted in a 6-nm layer located in the GaAs spacer layer 10 nm beneath each QD layer, and the doping concentration was controlled to be 25 acceptors per dot. The cross-sectional transmission electron microscopy (TEM) image of the InGa/GaAs QD layers is shown in Fig. 1a. The density of InAs/GaAs QDs is determined to be 4 × 10^10^ cm^−2^ by atomic force microscope measurement. The RTA treatment was performed in a N_2_ ambient at the temperature of 700 °C for 45 s. The QD samples were protected by a GaAs proximity cap during the annealing process.

### Design, Fabrication, and Characterization of LC-DFB

The schematic diagram of the designed LC-DFB laser structure is shown in Fig. 1a. This design approach enables the fabrication of LC-DFB lasers just by one round epitaxial growth and reduces the aspect ratio in optical grating etching. The formation of the narrow ridge waveguide and its lateral coupled grating structure is divided into two processing steps, which is different from the traditional defining lithography process [1, 9, 10]. The fabrication of the laterally coupled grating requires shallow etching and that reduces the high aspect ratio in dry etching demanded by the traditional deep etching approach. Moreover, etching the gratings only over a hundred nanometers into the semiconductors allows the grating structure with very small feature sizes like the first-order grating to be easily realized and hence provide a new opportunity to develop ingenious device structure towards THz applications.

Referring to the coupling principle of LC-DFB, it is well known that the proximity of the gratings to the ridge is a key factor that greatly influences the laser performance [20]. In the fabrication process, after the ridge waveguide is first defined, the sample for electron-beam lithography (EBL) has a height difference with respect to the waveguide, and the photoresist will stack aside the sidewall during EBL, which makes it difficult to make the formation of grating adjacent to the ridge. In order to solve the problem of the nonuniform photoresist coating and to form a high-quality grating patterned by EBL, the thickness of polymethylmethacrylate (PMMA) resist was carefully selected to be as thin as 75 nm, which is optimized to enable the grating quality to reach their equilibrium points. The LC-DFB laser was fabricated through the following procedures. First, a 75-nm SiO_2_ layer was deposited on top of the epitaxy structure by using plasma-enhanced chemical vapor deposition (PECVD), which acts as the etching protecting layer for the shallow etching of gratings. The ridge waveguide structure was patterned using optical lithography and etched to a depth of around 1.75 μm with the technique of inductively coupled plasma (ICP) with a gas mixture of Cl_2_ and BCl_3_. With the waveguide structure having been defined, the upper p-side AlGaAs cladding layer was further etched by wet etching which was stopped at~ 280 nm above the QD active regions. After that, the sample was spin-coated with PMMA resist (molecular weight of 950 K and thickness of 75 nm) and baked for 90 s at 180 °C. The first-order grating was defined alongside the ridge waveguide by EBL, and then the resist image was transferred into the AlGaAs by ICP dry etching. The etch rates of PMMA resist and AlGaAs were approximately 5 nm/s and 10 nm/s, respectively. A scanning electron microscopy (SEM) image of the fabricated LC-DFB structure is shown in Fig. 1b. Benefitting from the careful choice of the EBL exposure dose and the greatly alleviated photoresist stacking due to the thin resist, the gratings are tightly linked to the laser ridge waveguide, as revealed by the inset of Fig. 1b. The etching depth of grating is 135 nm, and the grating period is 194 nm. To achieve a precisely and widely tuned dual-wavelength lasing, two different Bragg periods were fabricated for lateral gratings at the two sides of the ridge waveguide. The Ohmic contact layer on the ridge waveguide was completely protected by the 75-nm-thick SiO_2_ protecting layer to ensure Ohmic contact surviving during the ICP etching process. The shallow-etched grating was controlled to be 150 nm above the QD active range to form a good coupling with light. For the aim of insulation and planarization, another layer of SiO_2_ was deposited on the sample with PECVD after etching the gratings. Finally, reactive ion etching (RIE) dry etching was employed to open a contact window in SiO_2_. Ti/Au and Au/Ge/Ni/Au were then deposited to form the top and bottom Ohmic contacts, respectively. The substrates were thinned down to around 80 μm to minimize the self-heating effect. The laser cavities of 1 and 0.45 mm long were fabricated, and the emitting facets were not coated. The laser bars were mounted with the p-side up on a copper heat sink, and all the measurements were performed under CW operation.

## Results and Discussion

**Fig. 2:**
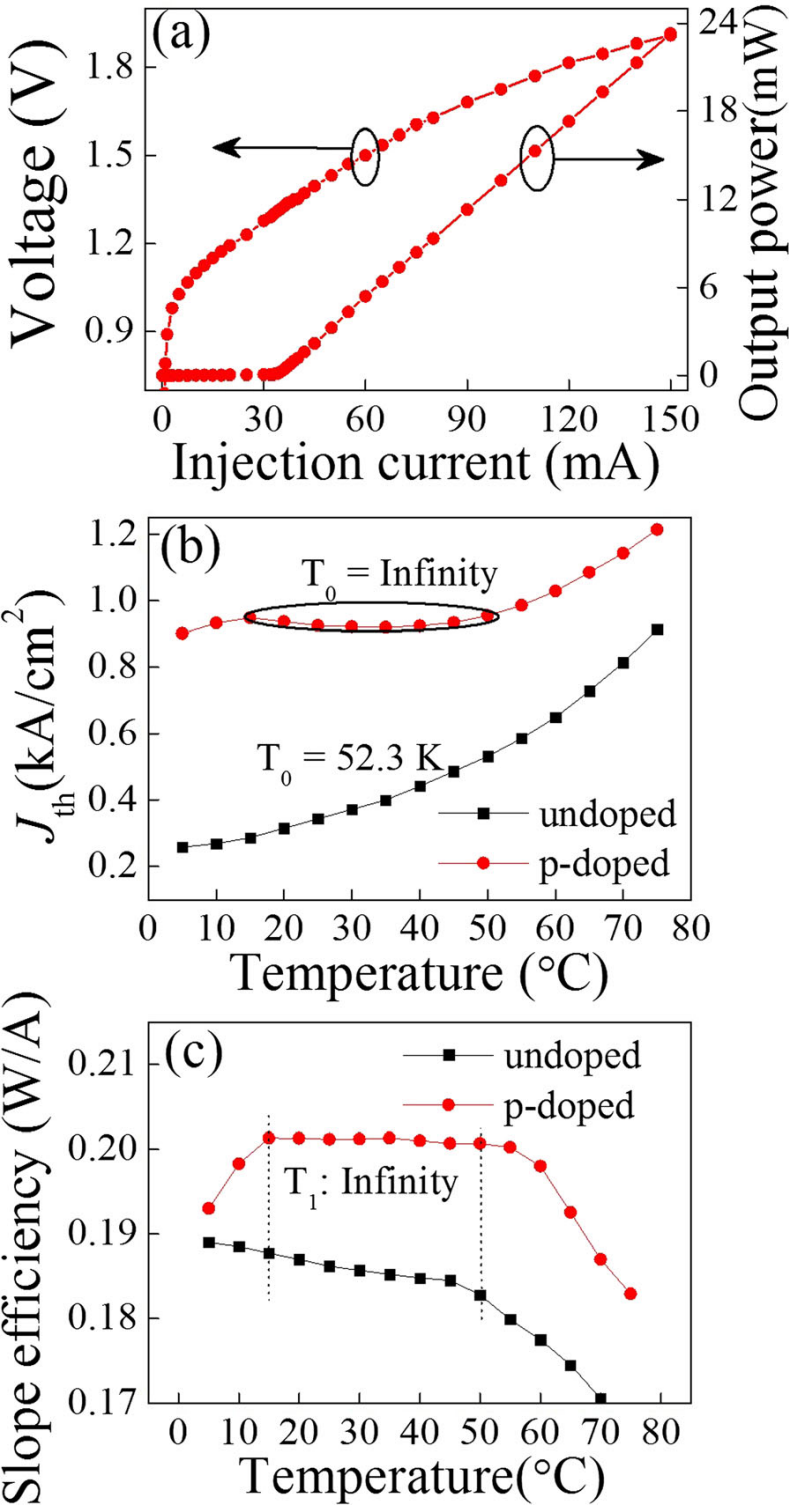
P–I–V and temperature dependence characteristic of the LC-DFB laser. **a** P–I–V characteristic of the p-doped LC-DFB laser at RT. **b** Temperature dependences of threshold current density for undoped and p-doped LC-DFB lasers. **c** Temperature dependences of slope efficiency for undoped and p-doped LC-DFB lasers

Figure 2a shows a typical power–current–voltage (P–I–V) characteristic of the as-fabricated LC-DFB laser based on a multiple modulation p-doped QD structure. The laser shows an obvious high slope efficiency of 0.20 W/A and a low threshold of 33 mA, which reveals the high material quality and high optical gain of the QD structure. The threshold current density and slope efficiency with respect to the temperature for an undoped and a p-doped QD LC-DFB lasers are presented in Fig. 2b, c, respectively. The characteristic temperature for the threshold current density (*T*_0_) of 52.3 K is calculated for the undoped QD LC-DFB laser as seen in Fig. 2b, while the *T*_0_ for p-doped QD LC-DFB laser has a significant increase, especially in the temperature range from 15 to 50 °C, in which an infinite *T*_0_ is observed. Moreover, in this temperature range, the slope efficiency almost shows no degradation (2.6% degradation for the undoped QD LC-DFB laser), indicating an infinite characteristic temperature for the slope efficiency (*T*_1_) for p-doped LC-DFB laser as well. The big difference of both *T*_0_ and *T*_1_ between the undoped and p-doped LC-DFB lasers is mainly attributed to the effects induced by the built-in excess holes due to the modulation p-doping which can significantly inhibit holes’ thermal broadening in the closely spaced energy levels [21, 22]. Based on the above results, the p-doped QD LC-DFB laser was selected for the further lasing spectra characterization.

**Fig. 3:**
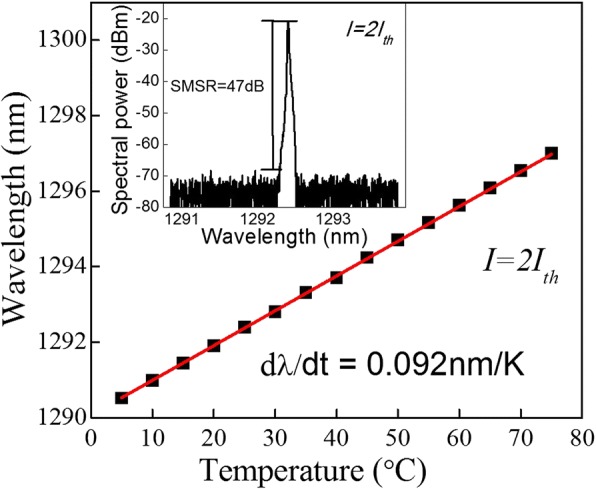
Temperature dependence of emission wavelength. Inset: emission spectrum of the p-doped LC-DFB laser measured at 2*I*_th_

The inset of Fig. 3 shows an emission spectrum of the p-doped LC-DFB laser of 1 mm in cavity length measured under *I* = 2*I*_th_ injection level at room temperature (RT), and a single longitudinal mode lasing at 1292.4 nm with a very large SMSR of 47 dB can be observed. Figure 3 shows the emission wavelength as a function of the operation temperature of the p-doped LC-DFB laser, which reveals a variation rate of only 0.092 nm/K. The high-temperature stability of the lasing wavelength is in good accordance with the temperature coefficient of the refractive index, which is about five times lower than that of the material gain shift.

Recently, Goshima et al. [10] reported a 1.3-μm InAs/GaAs QD LC-DFB laser fabricated by deep gratings vertically etched into the ridge waveguide structure, and a low slope efficiencies below 0.03 W/A and a small SMSR of 20 dB were observed, which are mainly due to the large waveguide losses caused by the deep etching process. With a shallow-etched grating structure, Briggs et al. [23] have successfully fabricated GaSb-based LC-DFB lasers with a larger SMSR of 25 dB. But further improvement was limited by the lower coupling coefficient due to the large distance between the gratings and the ridge waveguide, which is crucial for the performance of a LC-DFB laser. In our work, the narrow ridge waveguide and the grating structures were fabricated separately, resulting in a very sharp and smooth sidewall of the ridge waveguide and therefore little waveguide loss. The shallow etching method for the grating fabrication employed in our experiments can sharply reduce the aspect ratio of the etched gratings and allow the making of the high-quality first-order grating structure which ensures good coupling with light. By carefully controlling the thickness of the PMMA resist and the EBL lithography parameters, the stacking phenomena of photoresist aside the sidewall of ridges were effectively alleviated, which leads to the formation of gratings tightly adjacent to the laser ridge waveguide. In addition, the high dot density of ~ 4.3 × 10^10^ cm^−2^ obtained by optimizing the MBE epitaxy growth parameters and the high gain of the QD assemblies realized by the modulation p-doping and the post-growth annealing treatment may account for the large 47 dB SMSR of our LC-DFB laser.

Besides the widespread applications already demonstrated in long-distance optical transmission and wavelength division multiplex (WDM) systems due to the superior features of the narrow emission spectrum and high thermal stability, the LC-DFB lasers have also demonstrated advantages for generating CW THz radiation. Compared with the traditional method to create THz radiation by using two independent diode lasers [24–26], LC-DFB lasers with simultaneous emission of two modes are very attractive for fabricating THz radiation sources due to the cost-effectiveness, compactness, high stability, and high spectral quality. In contrast to quantum well (QW) lasers, QD-based emitters are well suited for broadband tunable sources owing to two unique features of QD structures. Firstly, the nature of low density of states leads to the easy saturation of the GS levels, resulting in the further population of the excited states (ES). Secondly, the dot size variation can be utilized to extend the tuning range, due to the fact that the wide size distribution of the self-assembled QD ensemble leads to a wide spectrum of light emission governed by the quantum size effect.

**Fig. 4:**
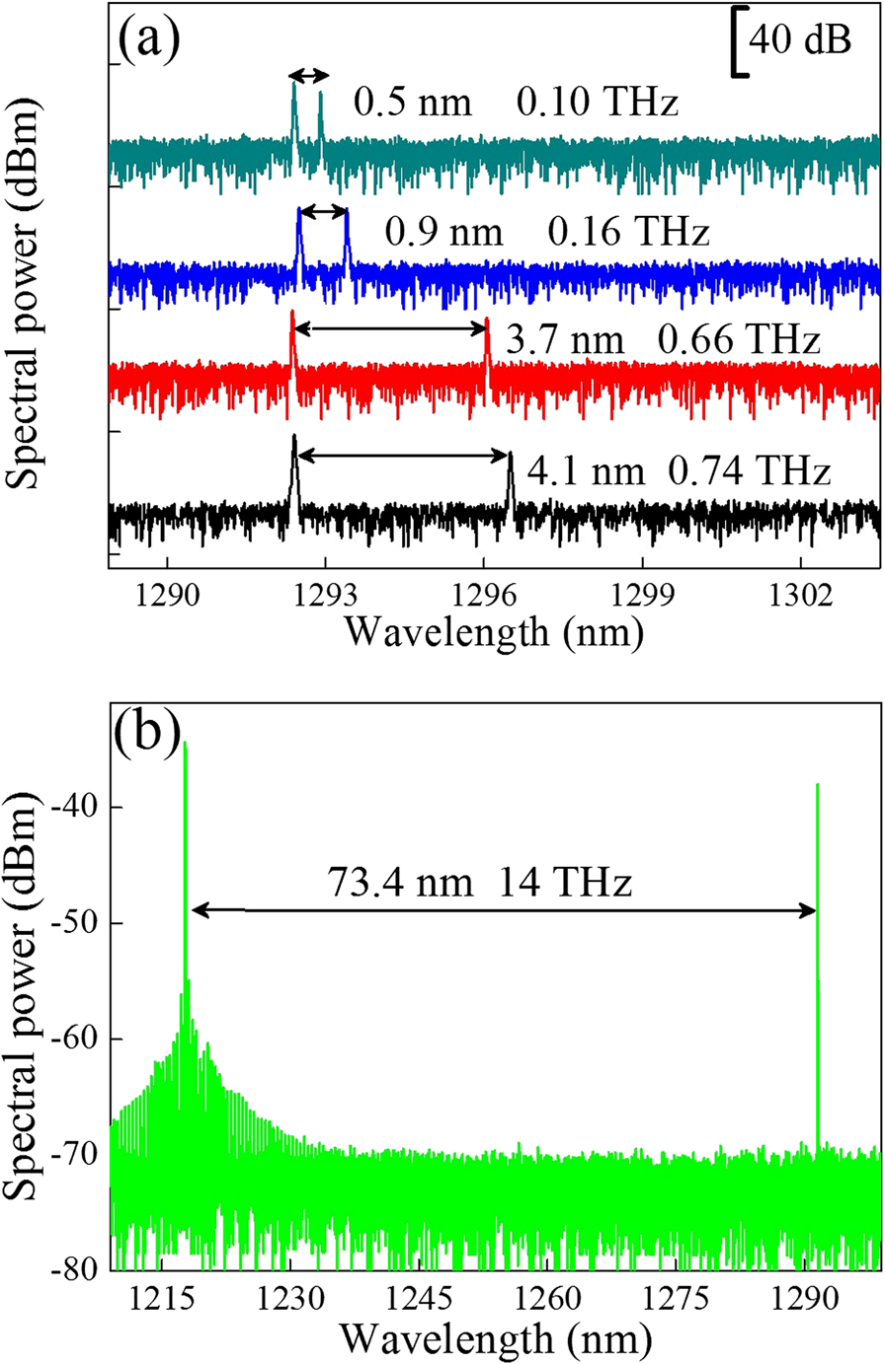
The spectrum of the dual-mode LC-DFB laser. **a** Emission spectra of the dual-wavelength LC-DFB laser with a different grating period. **b** Wide spacing of dual-mode lasing spectra of the LC-DFB laser with an ultra-short cavity length of 450 μm

The LC-DFB structure comprised of lateral gratings fabricated independently allows high flexibility in defining the designed Bragg wavelength. Dual-wavelength lasing can be achieved by fabricating two sets of gratings of different Bragg periods *Ʌ*_1_ and *Ʌ*_2_ which enable two different wavelengths *λ*_1_ and *λ*_2_. The method reported here involves defining two different grating periods for each side of the gratings. The dual-wavelength lasing measurements were carried out under CW conditions. Stable dual-wavelength lasing, with SMSR around 40 dB, was observed. As illustrated in Fig. 4a, the dark cyan, blue, red, and black lines exhibit lasing spectra with two different lasing wavelengths. For a 1-mm LC-DFB laser with grating period difference *Ʌ*_1_ − Ʌ_2_ = 0.10 nm, the two lasing wavelengths are 1292.40 and 1292.90 nm, respectively, yielding a wavelength spacing of 0.50 nm corresponding to the frequency difference of ~ 0.10 THz. By tuning the grating period difference to 0.64 nm, the dual-wavelength spacing can be extended to 4.1 nm which corresponds to a beating frequency of 0.74 THz.

To get a larger tuning range of the dual-mode lasing, the cavity length of LC-DFB lasers was carefully shortened to 450 μm, which results in the simultaneous GS and ES lasing due to the effect of GS gain saturation and increased population of the ES. The LC-DFB laser structure consists of two different Bragg periods of 182 and 194 nm, respectively, which is similar to what was described in previous reports [27, 28]. As shown in Fig. 4b, the two longitudinal modes exhibit a large wavelength separation of 73.4 nm, corresponding to the frequency difference of 14 THz. By implementing two different period gratings laterally to a ridge waveguide and shortening the cavity length delicately to allow ES lasing, the InAs/GaAs QD-based laser diodes could emit dual lasing lines of very wide tunable wavelength spacing from 0.5 to 73.4 nm corresponding to 0.10–14 THz frequency difference. Compared with other types of proposed schemes of THz photomixing based on two separate lasers, our device offers the advantages of simple structure, compact size, low fabrication cost, and a very wide tuning range.

## Conclusions

A 1.3-μm QD LC-DFB laser with shallow-etched gratings has been fabricated, in which the complexity of overgrowth and the difficulties of deep-etching processes in general fabrication process of DFB laser are successfully avoided. Benefitting from the high material gain of QD samples prepared with modulation p-doping, RTA treatment, and optimized LC-DFB laser waveguide structure, the device exhibits a large SMSR of 47 dB and a high thermal stability dλ/dT of 0.092 nm/K. By the means of defining two different periods to the gratings at each side of the narrow ridge waveguide or shortening the laser cavity length, two lasing lines can be obtained simultaneously and the spacing between the two lasing wavelengths can be flexibly and largely tuned, which can be modified from 0.5 to 73.4 nm, corresponding to the frequency difference from 0.10 to 14 THz. It is noteworthy that this wide tuning range is realized in a single laser device, which has not been reported so far. These results demonstrate the promising application of LC-DFB lasers for generating CW THZ radiation.

## Abbreviations

CW: Continuous-wave
DFB: Distributed feedback
EBL: Electron-beam lithography
ES: Excited states
GS: Ground state
ICP: Inductively coupled plasma
LC-DFB: Laterally coupled distributed feedback
MOCVD: Metalorganic vapor chemical deposition
PECVD: Plasma-enhanced chemical vapor deposition
P–I–V: Power–current–voltage
PMMA: Polymethylmethacrylate
QD: Quantum dot
QW: Quantum well
RT: Room temperature
RTA: Rapid thermal annealing
SEM: Scanning electron microscopy
SMSR: Side-mode suppression ratio
TEM: Transmission electron microscopy
WDM: Wavelength division multiplex
